# The non-peptide thrombopoietin receptor agonist eltrombopag stimulates megakaryopoiesis in bone marrow cells from patients with relapsed multiple myeloma

**DOI:** 10.1186/s13045-015-0136-2

**Published:** 2015-04-16

**Authors:** Jee-Yeong Jeong, Michelle S Levine, Nirmalee Abayasekara, Nancy Berliner, Jacob Laubach, Gary J Vanasse

**Affiliations:** Division of Hematology, Brigham and Women’s Hospital, Harvard Medical School, Boston, MA USA; Department of Biochemistry, Kosin University College of Medicine, Busan, South Korea; Cancer Research Institute, Kosin University College of Medicine, Busan, South Korea; Department of Medical Oncology, Dana-Farber Cancer Institute, Boston, MA USA; Present address: Translational Clinical Oncology, Novartis Institutes for Biomedical Research, Inc., Cambridge, MA USA

**Keywords:** Eltrombopag, Megakaryocytes, Multiple myeloma, CD34, CD138

## Abstract

**Background:**

Thrombocytopenia is a significant problem in patients with relapsed or refractory multiple myeloma, precipitating a need for supportive platelet transfusions and necessitating decreases in delivered doses of chemotherapy. Eltrombopag is a non-peptide, small molecule thrombopoietin (TPO) receptor agonist that promotes megakaryopoiesis similar to endogenous human TPO and may be an effective agent for thrombocytopenia in this patient population.

**Methods:**

We examined the effects of eltrombopag on megakaryocyte colony-forming capacity in CD34+ cells in patients with multiple myeloma and investigated its impact on proliferation, viability, and apoptosis in primary CD138+ human myeloma cells and myeloma cell lines.

**Results:**

Eltrombopag at doses of 0.1 to 100 μM did not enhance proliferation of primary human CD138+ multiple myeloma cells from patients with relapsed disease or myeloma cell lines when used alone or in combination with erythropoietin (EPO) and granulocyte colony-stimulating factor (G-CSF) and did not alter cell viability nor apoptosis of human myeloma cells exposed to bortezomib and lenalidomide. Eltrombopag stimulated megakaryopoiesis in human CD34+ cells from normal individuals and from patients with relapsed multiple myeloma via activation of Akt signaling pathways.

**Conclusions:**

These results provide proof-of-principle supporting the design of future clinical studies examining eltrombopag for the treatment of thrombocytopenia in patients with advanced multiple myeloma.

## Background

Clinically significant thrombocytopenia is a common problem in patients with relapsed or refractory multiple myeloma, resulting in increased bleeding complications, administration of supportive platelet transfusions, and necessitating reductions in delivered doses of chemotherapy. The etiology of thrombocytopenia in this patient population is multifactorial, often involving cytotoxic effects of chemotherapeutic agents on megakaryocytes, suppression of normal hematopoiesis by malignant cells, and cumulative disease- and therapy-related abnormalities affecting the bone marrow stromal microenvironment. Agents such as bortezomib, a potent reversible proteasome inhibitor [1], have been shown to be highly active in patients with relapsed or refractory multiple myeloma [2,3]. However, the most common grade 3 adverse event noted during clinical trials with bortezomib was cyclic thrombocytopenia, occurring in 24% and 28% of trial participants [4,5], potentially limiting intensity of dosing regimens.

Thrombopoietin (TPO) is synthesized in the liver and is the primary regulator of megakaryocyte development and platelet formation and is the ligand for the c-mpl cytokine receptor [6,7], which is present on the surfaces of cells of the megakaryocytic lineage as well as early-stage hematopoietic progenitors [8,9]. TPO-mediated c-mpl activation promotes not only the proliferation and differentiation of megakaryocytic progenitors but also enhances the viability of pluripotent stem cells and early progenitor cells of all hematopoietic lineages [10,11]. Recombinant human TPO (rhTPO) was previously shown to attenuate carboplatin-induced severe thrombocytopenia and reduce the need for supportive platelet transfusions in patients with gynecologic cancer [12]. However, clinical development of rhTPO for the treatment of chemotherapy-associated thrombocytopenia was discontinued due to the formation of neutralizing anti-TPO antibodies and subsequent development of thrombocytopenia associated with the use of pegylated recombinant human megakaryocyte growth and development factor (PEG-rHuMGDF) [12].

Eltrombopag is an orally bioavailable, non-peptide, small molecule c-mpl agonist that has been shown in both *in vitro* and *in vivo* studies to promote megakaryocyte proliferation and differentiation in a manner similar to that seen with endogenous human TPO [13]. Eltrombopag received accelerated FDA approval in the United States for the treatment of patients with chronic idiopathic thrombocytopenic purpura (ITP) in 2008 and full approval in 2011. Eltrombopag has been shown to effectively increase platelet counts and reduce thrombocytopenia-associated complications in patients with ITP and hepatitis C [14-16]. In addition, preclinical studies evaluating the effects of eltrombopag on bone marrow cells from patients with myelodysplastic syndrome (MDS) or acute myeloid leukemia (AML) found that it promoted normal megakaryopoiesis without inducing clonal expansion of malignant cells [17].

In this study, we addressed whether eltrombopag may promote megakaryopoiesis in bone marrow progenitors of patients with relapsed multiple myeloma without inducing proliferation of multiple myeloma cells or inhibiting immunomodulatory drug cytotoxicity. We found that eltrombopag did not stimulate the proliferation nor enhance the cell viability of human myeloma cell lines or primary CD138+ myeloma cells and did not alter drug-induced apoptosis of myeloma cells in patients with relapsed disease. Furthermore, we show that eltrombopag promotes megakaryopoiesis in CD34+ cells isolated from myeloma patients and healthy controls via activation of Akt signaling pathways, providing preclinical proof-of-principle to support the design of future clinical trials examining eltrombopag for the treatment of thrombocytopenia in patients with relapsed multiple myeloma.

## Results

### Multiple myeloma cells do not express MPL

**Figure 1:**
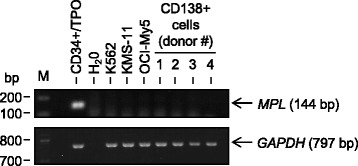
Human multiple myeloma cells do not express *MPL*. cDNA (10 ng/sample) prepared from primary CD138+ multiple myeloma cells (four representative patient samples) and from the KMS-11 and OCI-My5 human myeloma cell lines was used as a template to amplify a specific 144 bp fragment of the human *MPL* gene and a 797 bp fragment of the *GAPDH* gene by RT-PCR. cDNA prepared from normal CD34+ cells cultured in the presence of 100 ng/ml rhTPO for 4 days or K562 cells were used as positive and negative controls, respectively.

We examined whether c-mpl was expressed on human myeloma cell lines or primary CD138+ myeloma cells from patients with relapsed disease. Primary myeloma cells from each patient were found to be ≥95% CD138+/CD19−, as assessed by staining with CD138-PE and CD19-APC antibodies as previously described [18]. cDNA was prepared from the KMS-11 and OCI-My5 cell lines and from primary CD138+ myeloma cells from four subjects, and a specific 144 bp fragment of the human *MPL* gene and a 797 bp fragment of the *GAPDH* gene were amplified by PCR. cDNA prepared from normal CD34+ cells cultured in the presence of 100 ng/ml rhTPO for 4 days or K562 cells [19] were used as positive and negative controls, respectively. As shown in Figure 1, *MPL* gene expression was not detected in multiple myeloma cell lines or in primary CD138+ myeloma cells, suggesting that eltrombopag would be unlikely to stimulate the growth of human myeloma cells via activation of c-mpl-dependent signaling pathways.

### Eltrombopag does not enhance the proliferation of human multiple myeloma cell lines

**Figure 2:**
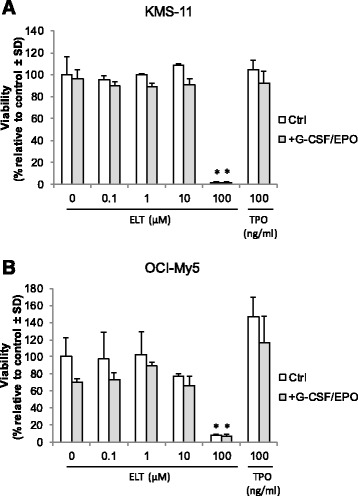
Eltrombopag does not enhance proliferation of human myeloma cell lines. **(A)** KMS-11 or **(B)** OCI-My5 cells were incubated with varying concentrations of eltrombopag in the presence or absence of G-CSF (10 ng/ml) and EPO (3 U/ml) for 6 days and cell viability determined by measuring fluorescence signals at 535_Ex_/595_Em_ using the CellTiter Blue Assay. Data are presented as mean ± S.D. of triplicate experiments. *p < 0.02. ELT = eltrombopag; TPO = thrombopoietin; CTRL = control.

We next investigated whether eltrombopag affects the proliferative capacity of human myeloma cells via c-mpl-independent pathways, either alone or in combination with other hematopoietic growth factors such as granulocyte colony-stimulating factor (G-CSF) and erythropoietin (EPO), which are often used as supportive therapy to treat cytopenias associated with anti-myeloma therapy. Proliferation of KMS-11 and OCI-My5 cell lines was analyzed in the presence of varying concentrations of eltrombopag (0–100 μM) or 100 ng/ml rhTPO in the presence or absence of 10 ng/ml G-CSF and 3 U/ml EPO over a period of 6 days. We found that eltrombopag or rhTPO did not enhance the proliferation of both KMS-11 and OCI-My5 at all concentrations tested either alone or in combination with G-CSF and EPO (Figure 2A,B). Similar results were observed with incubating cells for 3 or 9 days (data not shown). We also noted that the 100 μM concentration of eltrombopag markedly inhibited the proliferation and cell viability of KMS-11 and OCI-My5 cells, which is in agreement with other studies showing cell cytostatic/cytotoxic effects associated with this high concentration of eltrombopag [20].

### Eltrombopag does not alter chemotherapy-induced apoptosis of multiple myeloma cell lines

**Figure 3:**
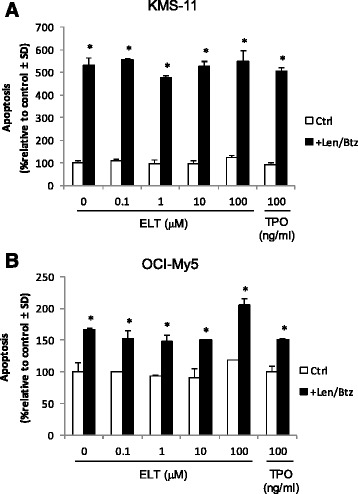
Eltrombopag does not inhibit bortezomib or lenalidomide-induced apoptosis of human myeloma cell lines. **(A)** KMS-11 or **(B)** OCI-My5 cells were incubated with varying concentrations of eltrombopag in the presence or absence of 1 μM lenalidomide and 10 nM bortezomib for 24 h and apoptosis determined by measuring activation of caspase-3 and −7 activity. Data are presented as mean ± S.D. of triplicate experiments. *p < 0.02. ELT = Eltrombopag; TPO = thrombopoietin; CTRL = control; Len = lenalidomide; Btz = bortezomib.

Chemotherapy regimens incorporating the use of bortezomib [3,5] or lenalidomide [21] are increasingly becoming standard of care in the treatment of patients with relapsed multiple myeloma. Because of this, we wanted to ensure that eltrombopag did not inhibit drug-induced apoptosis of human myeloma cell lines. KMS-11 and OCI-My5 cells were grown in the presence of varying concentrations of eltrombopag (0–100 μM) or 100 ng/ml rhTPO in the presence or absence of 1 μM lenalidomide and 10 nM bortezomib and apoptosis determined via measurement of caspase-3 and −7 activation at 24 h. The combination of lenalidomide and bortezomib induced 5.3-fold and 1.7-fold increases in apoptosis in KMS-11 and OCI-My5 cells, respectively, compared to control, and the degree of apoptotic activity was not altered by the presence of eltrombopag or rhTPO at all concentrations tested (Figure 3A,B).

### Eltrombopag does not enhance proliferation or alter chemotherapy-induced apoptosis of primary human CD138+ myeloma cells

**Figure 4:**
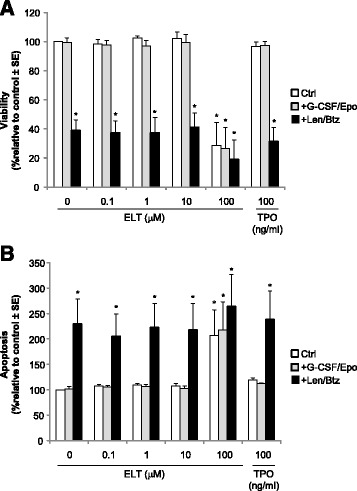
Eltrombopag does not enhance proliferation or alter apoptosis of primary human CD138+ myeloma cells. Human primary CD138+ multiple myeloma cells from patients with relapsed multiple myeloma (*n* = 7) were incubated with varying concentrations of eltrombopag in the presence or absence of G-CSF (10 ng/ml) and EPO (3 U/ml) and in the presence or absence of lenalidomide (1 μM) and bortezomib (10 nM). **(A)** Cell viability was determined by measuring fluorescence signals at 535_Ex_/595_Em_ using the CellTiter Blue Assay (*p < 0.01). **(B)** Apoptosis was assessed by measuring activation of caspase-3 and −7 (*p < 0.05). Data are presented as mean ± S.E. of combined experiments on seven individual patient samples, each performed in triplicate. ELT = Eltrombopag; TPO = thrombopoietin; CTRL = control; Len = lenalidomide; Btz = bortezomib.

We next evaluated eltrombopag’s effects on primary human myeloma cells. Equal numbers (5,000/well) of CD138+/CD19− myeloma cells isolated from bone marrow of patients with relapsed multiple myeloma (*n* = 7) were cultured in the presence of varying concentrations of eltrombopag (0–100 μM) or 100 ng/ml rhTPO in the presence or absence of 10 ng/ml G-CSF and 3 U/ml EPO for 24 h and then exposed to 1 μM lenalidomide and 10 nM bortezomib for an additional 24 and 48 h to measure apoptosis and cell proliferation, respectively. Eltrombopag at concentrations up to 10 μM (0.1–10 μM) or 100 ng/ml rhTPO did not alter cell viability in the presence or absence of G-CSF and EPO (Figure 4A). Cell proliferation studies revealed that the addition of 1 μM lenalidomide and 10 nM bortezomib resulted in 60% decreased cell viability of CD138+ myeloma cells, and this decreased viability was not inhibited by eltrombopag or rhTPO (Figure 4A). Apoptosis of CD138+ myeloma cells was not altered by either eltrombopag at concentrations up to 10 μM or 100 ng/ml rhTPO in the presence or absence of G-CSF and EPO (Figure 4B). Cells grown in the presence of lenaldomide and bortezomib exhibited 2.3-fold greater apoptosis, and this was again not altered by either eltrombopag or rhTPO (Figure 4B). We found that 100 μM eltrombopag enhanced apoptosis, resulting in markedly decreased CD138+ cell viability (Figure 4A,B).

### Eltrombopag stimulates the *ex vivo* expansion of hematopoietic progenitors

**Figure 5:**
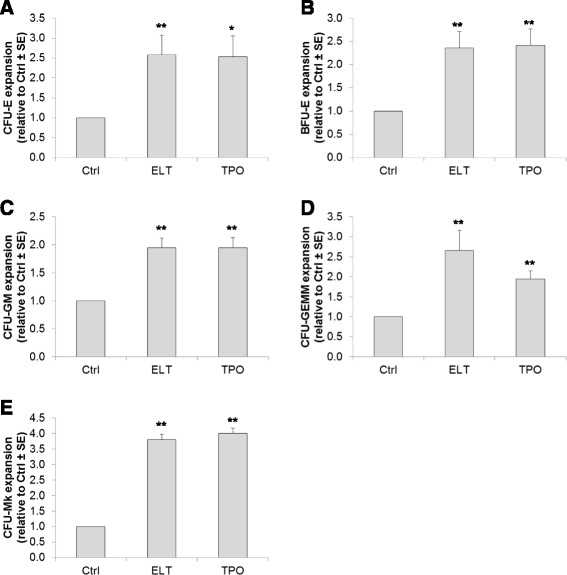
Eltrombopag stimulates the expansion of hematopoietic progenitor cells from normal controls. CD34+ cells cultured in the presence or absence of 10 μM eltrombopag or 100 ng/ml rhTPO for 11 days were assessed for pluripotency by hematopoietic progenitor assays performed in semi-solid methylcellulose or MegaCult media. **(A)** CFU-E, **(B)** BFU-E, **(C)** CFU-GM, and **(D)** CFU-GEMM were enumerated at the end of 14 days and **(E)** CFU-Mk at the end of 12 days. Data are presented as mean ± S.E. relative to control from eight individual donors with experiments performed in triplicate. CTRL = control; ELT = Eltrombopag; TPO = thrombopoietin. ^*^ *p* < 0.02, ^**^ *p* < 0.01.

We determined whether eltrombopag, in combination with other growth factors and early-acting cytokines, would support the expansion of human hematopoietic progenitors to an equivalent degree as that seen with rhTPO. Bone marrow-derived CD34+ cells from normal healthy controls (*n* = 8) were cultured in StemSpan medium supplemented with rhSCF (50 ng/ml), rhIL-3 (10 ng/ml), and rhIL-6 (10 ng/ml) in the presence or absence of 10 μM eltrombopag or 100 ng/ml rhTPO for 11 days, and hematopoietic progenitor assays were performed as described. CD34+ cells cultured in the presence of 10 μM eltrombopag were noted to have significant expansion of all hematopoietic colonies, including increases of 2.6-fold in CFU-E (*p* < 0.01), 2.4-fold in BFU-E (*p* < 0.01), 2.0-fold in CFU-GM (*p* < 0.01), and 2.7-fold in CFU-GEMM (*p* < 0.01) colonies compared to control (Figure 5A–D). There did not appear to be a statistically significant difference in hematopoietic colony expansion between eltrombopag and rhTPO except for CFU-GEMM, where eltrombopag appeared superior to rhTPO (2.7- versus 1.9-fold; *p* < 0.01, *t*-test). To examine the effect of eltrombopag on megakaryocyte colony formation (CFU-MK), CD34+ cells cultured as above were similarly measured using standard MegaCult assays containing 50 ng/ml rhTPO. CD34+ cells cultured in the presence of 10 μM eltrombopag exhibited a 3.8-fold increase in total MK colonies compared to control cells (*p* < 0.01, *t*-test) and proved equivalent to 100 ng/ml rhTPO (Figure 5E).

### Eltrombopag stimulates megakaryopoiesis in CD34+ cells isolated from patients with relapsed multiple myeloma and from normal controls

**Figure 6:**
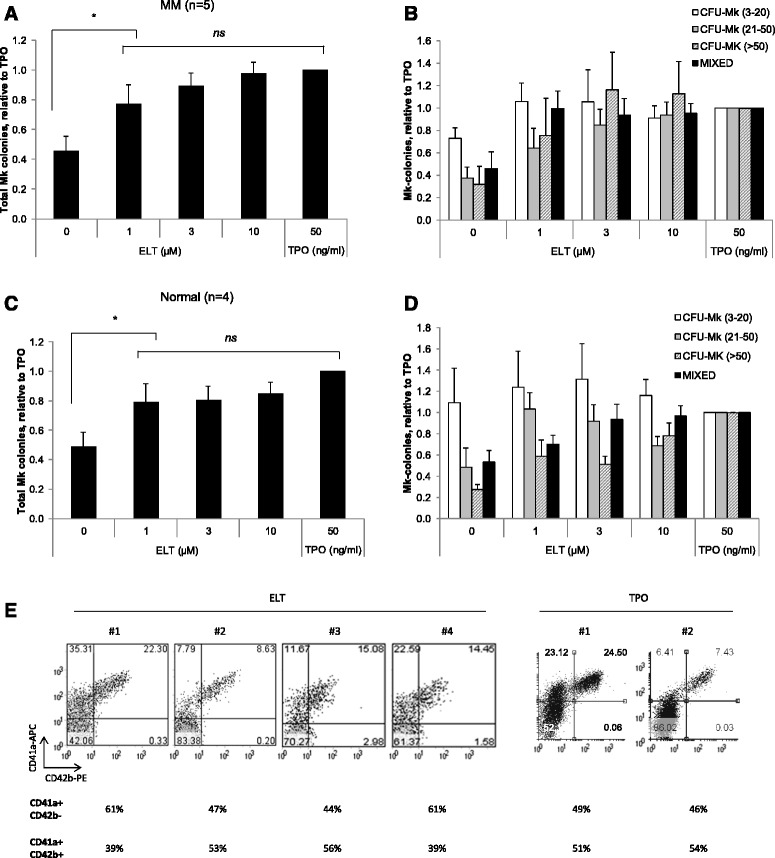
Eltrombopag stimulates megakaryopoiesis in CD34+ cells isolated from patients with relapsed multiple myeloma. CD34+ cells isolated from patients with relapsed multiple myeloma (*n* = 5, **A** and **B**) or CD34+ cells from normal controls (*n* = 4, **C** and **D**) were evaluated in CFU-Mk progenitor assays performed in semi-solid MegaCult media in the presence or absence of 0–10 μM eltrombopag or 50 ng/ml rhTPO. CFU-Mk colonies were enumerated after incubation for 11 days. Total CFU-Mk numbers are presented in **(A)** and **(C)**, and differential colony numbers by their size are presented in **(B)** and **(D)**. Data are presented as the mean Mk colony number relative to that with 50 ng/ml TPO (mean ± S.E., *n* = 5 individual patients or *n* = 4 normal controls with experiments each performed in triplicate). ^*^ *p* < 0.02 for all doses tested. ns = not significant. **(E)** CD34+ cells from normal controls (*n* = 4 for eltrombopag and *n* = 2 for rhTPO) were cultured in a serum-free StemSpan liquid medium containing rhSCF (50 ng/ml), rhIL-3 (10 ng/ml), and rhIL-6 (10 ng/ml) in the presence of 10 μM eltrombopag or 100 ng/ml rhTPO for 11 days, and the surface expression of CD41a and CD42b was analyzed by flow cytometry. Each dot plot represents individual donor.

We next investigated whether eltrombopag is capable of stimulating megakaryopoiesis in purified CD34+ cells isolated from patients with relapsed multiple myeloma (*n* = 5). In Mk progenitor assays, CD34+ cells cultured in the presence of varying concentrations of eltrombopag (1–10 μM) exhibited a significant increase in Mk colonies at all doses tested compared to control cells (*p* < 0.02, *t*-test) and proved equivalent to 50 ng/ml rhTPO (Figure 6A,B). Similar results were obtained with CD34+ cells isolated from normal controls (*n* = 4, Figure 6C,D). In liquid culture, the percentage of immature and mature megakaryocytes was assessed by fluorescence-activated cell sorting (FACS) analysis for CD41a and CD42b expression on cells cultured for 11 days [22]. Eltrombopag and rhTPO equivalently produced an immature population of megakaryocytes, with 53% being CD41a+/CD42b − and 47% being CD41a+/CD42b + in average among CD41a + cells incubated with eltrombopag and 47.5% being CD41a+/CD42b − and 52.5% being CD41a+/CD42b + when incubated with rhTPO (Figure 6E). In summary, our data support the ability of eltrombopag to stimulate megakaryopoiesis in hematopoietic progenitors from patients with relapsed multiple myeloma.

### Eltrombopag activates Akt in both human platelets and CD34+ cells

**Figure 7:**
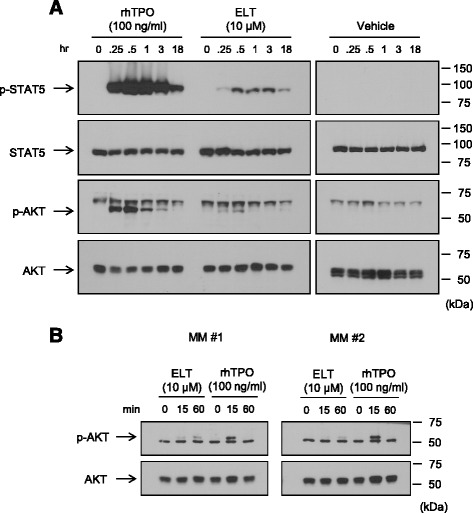
Eltrombopag induces Akt in human platelets and immature megakaryocytes. **(A)** Platelets from a healthy donor or **(B)** multiple myeloma-derived peripheral blood mobilized-CD34+ cells (MM#1 and MM#2) cultured for 8 days were stimulated with 100 ng/ml rhTPO or 10 μM eltrombopag for the indicated times, and immunoblotting was performed to detect the activation of STAT5 and Akt signaling pathways. Note that p-Akt bands appeared below non-specific bands in human platelets **(A)** and above non-specific bands in immature megakaryocytes **(B)**. Migration of molecular weight markers is indicated to the right of each blot.

Whereas both eltrombopag and rhTPO appear to activate STAT proteins, it has been previously reported that in contrast to rhTPO, eltrombopag-mediated signaling in human platelets did not involve Akt phosphorylation [23]. To determine eltrombopag’s effects on Akt signaling during megakaryopoiesis, we examined whether eltrombopag induces Akt phosphorylation in mature platelets and an earlier stage of megakaryocyte development. Contrary to previous reports, we found that eltrombopag induced Akt phosphorylation in human platelets, albeit to a lesser degree compared to rhTPO (Figure 7A). Next, peripheral blood-mobilized CD34+ cells isolated from two patients with relapsed multiple myeloma were cultured as described for 8 days and then cytokine-starved and stimulated with either eltrombopag or rhTPO. In this group of immature megakaryocyte progenitors, we found that both eltrombopag and rhTPO induced Akt signaling, with eltrombopag exhibiting less intense and delayed Akt phosphorylation compared to rhTPO (Figure 7B). Similar degrees of Akt phosphorylation in response to eltrombopag and rhTPO were also noted in peripheral blood-mobilized CD34+ cells from normal, healthy individuals (data not shown).

## Discussion

In this study, we examined the effect of the novel non-peptide thrombopoietin receptor agonist eltrombopag on human multiple myeloma cell lines and on bone marrow-derived CD138+ myeloma cells and hematopoietic progenitors in patients with relapsed multiple myeloma. Eltrombopag received accelerated FDA approval for the treatment of patients with chronic ITP in 2008 and full approval in 2011. A preclinical study reveals it may support megakaryopoiesis in patients with MDS/AML [17]. A recent article reviewed the efficacy and safety of thrombopoietin receptor agonists including eltrombopag for the past 5 years since the FDA approval, summarizing that eltrombopag effectively increases platelet counts with few adverse effects [24]. The effects of eltrombopag on patients with multiple myeloma or other lymphoid malignancies have not been previously reported. Thrombocytopenia remains an important clinical consideration in multiple myeloma, especially in those patients with relapsed or refractory disease [4,5]. Although multiple myeloma remains an incurable malignancy, the introduction of novel therapeutic agents has significantly improved clinical outcomes in a large number of patients [25,26]. As clinically significant thrombocytopenia may predispose to bleeding and may necessitate the need for dose reductions in chemotherapy, patients would benefit from an agent that will stimulate platelet production and thus reduce the need for supportive platelet transfusions and minimize chemotherapy dose modifications. Our study describes for the first time the effects of eltrombopag in multiple myeloma. We found no evidence to suggest that eltrombopag promotes proliferation of human multiple myeloma cell lines or primary CD138+ myeloma cells, and it does not appear to inhibit the cytotoxic effects of bortezomib or lenalidomide. Furthermore, we show that eltrombopag is capable of supporting megakaryopoiesis in hematopoietic progenitors from patients with relapsed multiple myeloma via pathways that involve Akt activation. These results provide compelling preclinical evidence that eltrombopag may be a useful agent for the treatment of thrombocytopenia in some patients with relapsed or refractory multiple myeloma and warrants further investigation in controlled clinical trials.

We used several independent assays to determine whether eltrombopag had the capacity to promote proliferation of multiple myeloma cells. As *MPL* gene expression pattern may vary according to the malignant cell of origin, being found in MDS/AML but not in acute lymphoblastic leukemia (ALL) or non-Hodgkin’s lymphoma (NHL) [19,27], we initially investigated whether *MPL* was expressed in human primary myeloma cells and cell lines. We did not detect *MPL* expression in primary CD138+ myeloma cells and myeloma cell lines by PCR analysis, making it unlikely for eltrombopag to promote *in vitro* or *ex vivo* proliferation of multiple myeloma cells via c-mpl-dependent pathways. More importantly, we found that eltrombopag did not enhance the proliferative capacity of primary human CD138+ myeloma cells or myeloma cell lines at all doses tested. Eltrombopag has been shown to reduce proliferation of non-megakaryocytic leukemia and lymphoma cell lines [20]. However, we could not detect an anti-proliferative effect of eltrombopag on the myeloma cell lines we examined. Although the 100 μM dose of eltrombopag markedly decreased myeloma cell viability, this likely reflects a general cell cytotoxic effect rather than a specific anti-proliferative effect, as this high dose was also noted to suppress the development of normal hematopoietic progenitor colonies.

The cumulative effect of chemotherapy in patients with relapsed or refractory multiple myeloma may result in progressive bone marrow suppression and complications due to anemia, neutropenia, and thrombocytopenia. The appropriate use of erythropoiesis-stimulating agents (ESAs) for the treatment of chemotherapy-induced anemia is currently being reevaluated. Although the use of ESAs and G-CSF may have a beneficial effect in patients with MDS [28,29], their benefit in multiple myeloma patients remains in question [30,31]. Nevertheless, given that growth factors may be prescribed for some patients with multiple myeloma, we wanted to determine whether eltrombopag may synergistically interact with EPO and G-CSF to promote proliferation of human myeloma cells. Our results demonstrate that eltrombopag, in combination with EPO and G-CSF, did not enhance the proliferation of primary human CD138+ myeloma cells or myeloma cell lines. Taken together, these preclinical results indicate that eltrombopag at all doses studied does not enhance the proliferative capacity of primary human CD138+ myeloma cells from patients with relapsed disease or myeloma cells lines. Eltrombopag was also found not to abrogate the apoptotic activity or cytotoxicity of either bortezomib or lenalidomide, an important consideration given the widespread use of these agents in patients with relapsed multiple myeloma and increased administration as front-line therapy. However, due to the limited number of patient samples examined in our study, we cannot say that eltrombopag may not adversely affect the proliferation of multiple myeloma in particular subgroups of patients and does not take into account the potential for deleterious interactions with the bone marrow microenvironment. Therefore, our data will need to be further analyzed and confirmed in larger numbers of patients to determine whether eltrombopag will prove to be a safe and efficacious treatment for thrombocytopenia in subgroups of multiple myeloma patients. Other safety concerns such as thrombosis risk and fibrosis risk in multiple myeloma patients should be considered as well. Based on clinical trials of eltrombopag in other diseases, these risks appeared to be minimal to negligible unless longer than 2–3 years of treatment is required [24]. Therefore, it is highly unlikely that eltrombopag increases the risk for thrombosis and/or fibrosis since eltrombopag would only be used on an intermittent basis to support platelet counts in multiple myeloma patients before or after chemotherapy. Nevertheless, potential adverse effects should be fully evaluated in future clinical trials.

We show for the first time that eltrombopag is capable of stimulating megakaryopoiesis in CD34+ cells isolated from patients with relapsed multiple myeloma and did so to a degree equivalent to that of rhTPO. The use of rhTPO or megakaryocyte-derived growth factor (MDGF) for the treatment of thrombocytopenia in multiple myeloma patients has not been previously evaluated. rhTPO, when used in combination with other cytokines, has been shown to support the *ex vivo* expansion of megakaryocyte progenitors from normal bone marrow and peripheral blood and from patients with hematological malignancies [32]. In patients undergoing autologous stem cell transplantation (ASCT) following high-dose chemotherapy, combined administration of G-CSF and rhTPO enhanced the mobilization of CD34+ hematopoietic progenitors, resulting in a statistically significant reduction in time to neutrophil recovery as well as reduction in the number of platelet and red blood cell transfusions [33]. However, the clinical utility of rhTPO was limited by drug-associated immunogenicity in humans [34], necessitating the discontinuation of clinical development of these agents. Our study shows that eltrombopag supports the expansion of human hematopoietic progenitors to an equivalent degree as that seen with rhTPO. Both eltrombopag and rhTPO also favored the development of more immature megakaryocytic progenitors, as evidenced by greater cell surface expression of CD41a (glycoprotein IIb/IIIa) and relative lack of expression of CD42b (glycoprotein Ib), which is a similar pattern to that noted previously with rhTPO [22]. Contrary to a previous report [23], we found that eltrombopag induces activation of Akt in both human platelets and immature megakaryocytes but does so to a lesser degree and with different kinetics compared to rhTPO. Our finding may reflect greater sensitivity of presently available Akt antibodies or biological differences in patient samples examined. The biological or clinical significance of eltrombopag-mediated Akt activation in patients with lymphoid malignancies remains to be determined. Overall, our data suggest that eltrombopag may prove to be a clinically useful alternative to rhTPO for stimulating megakaryopoiesis in relapsed multiple myeloma patients.

## Conclusions

Our preclinical study provides proof-of-principle demonstrating that eltrombopag is capable of stimulating megakaryopoiesis in CD34+ cells from patients with relapsed multiple myeloma and does not promote proliferation of human primary multiple myeloma cells or myeloma cell lines. Furthermore, no synergistic effect on myeloma cell growth was noted with the combination of eltrombopag, EPO, and G-CSF, and eltrombopag did not appear to reverse the cytotoxic or apoptotic effects of lenalidomide or bortezomib. Insights garnered from this study will help form the basis for larger preclinical studies examining the effects of eltrombopag on hematopoiesis in multiple myeloma and provides a rationale to support the design of future clinical trials evaluating the use of eltrombopag for the treatment of thrombocytopenia in patients with relapsed multiple myeloma as well as other lymphoid malignancies.

## Methods

### Reagents

Eltrombopag was provided by GlaxoSmithKline. Eltrombopag was dissolved in sterile distilled deionized water at a 10 mM stock concentration and stored light-protected at room temperature and used within 2 weeks of preparation.

### Patient samples

Bone marrow (*n* = 4) and G-CSF-mobilized peripheral blood hematopoietic progenitor (*n* = 4) samples were obtained from patients with relapsed multiple myeloma within the framework of routine standard of care under the auspice of Dana-Farber Cancer Institute protocol (DFCI) 01–206 after obtaining written informed consent in accordance with the Declaration of Helsinki. Purified human CD138+ multiple myeloma cells (*n* = 3) or bone marrow mononuclear cells [BM-MNC (*n* = 2)] from bone marrow from patients with relapsed multiple myeloma were also obtained from commercial sources (AllCells, Emeryville, CA and Proteogenix, Oberhausberger, France, respectively). Cryopreserved bone marrow-derived (*n* = 8) or peripheral blood-mobilized (*n* = 2) human CD34+ cells from healthy controls were purchased from Lonza (Basel, Switzerland) and AllCells, respectively. Normal human platelets were obtained from the Transfusion Medicine Service at Brigham and Women’s Hospital (Boston, MA).

### Isolation of human CD138+ multiple myeloma and CD34+ cells

Bone marrow aspirates and mobilized peripheral blood progenitors were processed by density centrifugation using Ficoll-Hypaque to obtain BM-MNC and PB-MNC, respectively. CD138+ myeloma cells and CD34+ cells were purified using anti-CD138 and anti-CD34 immunomagnetic bead selection, respectively, by AutoMACS column purification (Miltenyi Biotec, Auburn, CA) according to the manufacturer’s instructions.

### Culturing primary multiple myeloma cells and cell lines

Human multiple myeloma cell lines KMS-11 and OCI-My5 were kindly provided by Dr. Constantine Mitsiades of DFCI and were maintained in RPMI-1640 (Invitrogen, Carlsbad, CA) containing 10% fetal bovine serum (FBS; Invitrogen) and 1 × antibiotic-antimycotic (AA; Invitrogen). Primary human CD138+ multiple myeloma cells were cultured for up to 72 h in RPMI-1640 with 20% FBS, 1 × AA, and the following combination of cytokines each at 10 ng/ml: interleukin six (rhIL-6), vascular endothelial growth factor (rhVEGF), insulin-like growth factor-1 (rhIGF-1), hepatocyte growth factor (rhHGF), and rhIL-13 (all from Peprotech, Rocky Hill, NJ). The K562 cell line was cultured in RPMI-1640 containing 10% FBS and 1 × AA. All cell cultures were maintained at 37°C in a humidified atmosphere of 95% air and 5% CO_2_.

### Hematopoietic progenitor cell assays

CD34+ cells were cultured in StemSpan serum-free expansion medium (StemCell Technologies) containing recombinant human stem cell factor (rhSCF, 50 ng/ml), interleukin-3 (rhIL-3, 10 ng/ml), rhIL-6 (10 ng/ml, all from Peprotech), and low-density lipoprotein (40 μg/ml, Sigma-Aldrich), in the presence or absence of eltrombopag (10 μM) or rhTPO (100 ng/ml, Peprotech) and 1 × AA for 11 days. Cell density was maintained in a range of 1 × 10^5^–2 × 10^6^ cells/ml by replenishing with fresh medium on days 4 and 7. Cultured cells were plated in triplicate in 35-mm dishes (5,000 total cells per dish) in a semisolid methylcellulose medium consisting of 80% methocult H4230 media (StemCell Technologies) and 20% IMDM (Invitrogen), supplemented with 50 ng/ml rhSCF, 10 ng/ml rhIL-3, 10 ng/ml granulocyte-macrophage colony-stimulating factor (GM-CSF; PeproTech), 1 U/ml erythropoietin (EPO; R&D Systems, Minneapolis, MN), and 1 × AA. Colony-forming unit-erythroid (CFU-E), burst-forming unit-erythroid (BFU-E), CFU-granulocyte/macrophage (CFU-GM), and CFU-granulocyte/erythrocyte/monocyte/megakaryocyte (CFU-GEMM) were scored using an inverted microscope (Nikon, Tokyo, Japan) 14 days following plating. Megakaryocytic colony (CFU-Mk) formation was assessed using the Megacult-C CFU-Mk assay kit (StemCell Technologies) according to the manufacturer’s instructions. Briefly, CD34+ cells were plated in triplicate in dual-chamber slides (DCS) (2,500 cells per DCS) in semisolid Megacult-C medium supplemented with 10 ng/ml rhIL-3 (PeproTech), 10 ng/ml rhIL-6 (Peprotech), and 40 μg/ml LDL (Sigma-Aldrich), in the presence or absence of varying concentrations of eltrombopag (0–10 μM) or 50 ng/ml rhTPO. Megakaryocyte colonies were scored by immunocytochemical stainings with anti-human GPIIb/IIIa (CD41a) antibody, biotin-conjugated goat anti-mouse IgG, avidin-alkaline phosphatase conjugate, and alkaline phosphatase substrate. CFU-Mk were defined as clusters of more than three CD41a + cells [35]. Colonies containing both CD41a + and CD41a − cells were scored as mixed colonies originating from the most immature progenitors capable of differentiating into erythroid and Mk cells.

### Flow cytometry

Cells were washed in 4°C in phosphate-buffered saline (PBS) containing 1% FBS and stained with the following human monoclonal antibodies: fluorescein isothiocyanate (FITC)-conjugated CD34 (BD Biosciences, San Diego, CA), phycoerythrin (PE)-conjugated CD138 (Miltenyi Biotec), allophycocyanin (APC)-conjugated CD19 (Miltenyi Biotec), APC**-**CD41a, and PE-CD42b. Non-viable cells were excluded by counter staining with 7-AAD (BD Biosciences). Isotype-matched FITC- or PE-conjugated antibodies were used as controls. Cells were analyzed on a FACScaliber cytometer (BD Biosciences) using Cellquest Pro Software (BD Biosciences) or Flowing Software (flowingsoftware.com).

### Reverse transcription polymerase chain reaction

Total RNA was isolated from cells using the RNAqueous-4 PCR kit (Invitrogen) or the RNAqueous-Micro kit (Invitrogen), including DNase I treatment, according to the manufacturer’s instructions. First-strand cDNA was generated by using the RETROscript kit (Invitrogen) with a random decamer primer. *MPL* gene expression was determined by end-point polymerase chain reaction (PCR) using the sense primer (5′-atgctagctcccaaggcttcttct) and the antisense primer (5′-acttgaagtggcagcgagagaact) with a modified touchdown PCR cycle: 94°C/5 min, 20 cycles of 94°C/30 s–65°C (−0.5°C per cycle)/30 s–72°C/30 s, 20 cycles of 94°C/30 s–55°C/30 s–72°C/30 s, 72°C/5 min. The levels of *GAPDH* in the same templates were determined by semi-quantitative PCR using the sense primer (5′-aaggctgagaacgggaagctt) and the antisense primer (5′-tccaccaccctgttgctgta) as above.

### Multiple myeloma cell proliferation and apoptosis assays

Equal numbers of either primary CD138+ myeloma cells (5,000 cells/well) or myeloma cell lines (1,000 cells/well) were plated in 96-well fluorescence/luminescence compatible plates (Corning, Corning, NY) in triplicate in the presence or absence of varying concentrations of eltrombopag (0.1–100 μM) or rhTPO (100 ng/ml) as well as in the presence or absence of G-CSF (10 ng/ml) and EPO (3 U/ml) for varying periods of time as described. Cell viability was determined using the CellTiter Blue Assay (Promega, Madison, WI) measuring fluorescence signals at 535_Ex_/595_Em_ according to the manufacturer’s instructions and confirmed by incorporation of 5-bromo-2-deoxyuridine (BrdU; BrdU in situ detection kit, BD Pharmingen, San Diego, CA).

To measure apoptosis, subsets of the plated cells cultured as above at concentrations of 5,000 cells/well for primary CD138+ myeloma cells and 10,000 cells/well for myeloma cell lines were treated with or without lenalidomide (1 μM) and bortezomib (10 nM). Apoptosis was determined 24 h post treatment with lenalidomide/bortezomib by measuring caspase-3 and −7 chemiluminescent activities using the Caspase-Glo 3/7 assay system (Promega) according to the manufacturer’s instructions. Fluorescence/luminescence signals were measured using a DTX880 Multimode Detector (Beckman Coulter, Brea, CA). All plates contained control wells with medium alone and additional control wells with medium plus eltrombopag. All sample data was processed with corresponding controls.

### Signal transduction experiments

Human platelets or CD34+ cells cultured in StemSpan medium containing 50 ng/ml rhSCF, 10 ng/ml rhIL-3, 10 ng/ml rhIL-6, 100 ng/ml rhTPO, and 1 × AA for 8 days were cytokine-starved in RPMI-1640 medium containing 0.5% FBS for 3 h. Cells were stimulated with either 10 μM eltrombopag or 100 ng/ml rhTPO for specified durations, and whole cell lysates were prepared with 2 × sodium dodecyl sulfate polyacrylamide gel electrophoresis (SDS-PAGE) sample buffer containing Protease Inhibitor Cocktail Set III (CalBiochem/EMD Chemicals, Gibbstown, NJ, USA) and Phosphatase Inhibitor Cocktail Set III (CalBiochem) and boiled for 5 min. Immunoblotting was performed using antibodies specific for signal transducers and activators of transcription (STAT)-5, phospho-STAT5, phospho-Akt, and Akt (all from Cell Signaling Technology, Danvers, MA).

### Statistical analysis

Data are presented as mean ± standard error (SE) or standard deviation (SD). Differences between two groups of data were analyzed using the Student’s *t*-test. The level of significance was set at *p* value less than 0.05.
